# Health Notes for Students

**Published:** 1890-09

**Authors:** 


HEALTH NOTES FOR STUDENTS. By BURT G. WILDER, B. S., M. D., Professor of Physiology, Comparative Anatomy, and Zoölogy in Cornell University. Second edition, revised and enlarged. New York and London: G. P. Putnam's Sons. 1890.

Brief and concise is this little volume, every page of which teems with nuggets of useful information for those about to enter upon their student career. It was first intended for students entering Cornell University, in lieu of a public lecture on hygiene, but has now many times outgrown the author's original purpose.

The writer takes the student into his confidence, and tells him how he can best regulate his daily habits and appetites to conform with the duties and cares of student life. The early instruction in hygiene, which is so sadly neglected in many households, is here set forth in that clear and pleasing style so characteristic of the author's writings. It would be well were this little book placed in the hands or every school-boy and school-girl old enough to know that a healthful virtue is necessary to a valetudinarian happiness. W. C. K.

